# The Activity of a Hexameric M17 Metallo-Aminopeptidase Is Associated With Survival of *Mycobacterium tuberculosis*

**DOI:** 10.3389/fmicb.2017.00504

**Published:** 2017-03-27

**Authors:** Andre F. Correa, Izabela M. D. Bastos, David Neves, Andre Kipnis, Ana P. Junqueira-Kipnis, Jaime M. de Santana

**Affiliations:** ^1^Laboratório de Interação Patógeno-Hospedeiro, Instituto de Biologia Universidade de BrasíliaBrasília, Brazil; ^2^Instituto de Patologia Tropical e Saúde Pública Universidade Federal de GoiásGoiânia, Brazil

**Keywords:** MtLAP, proteases, tuberculosis, leucine aminopeptidase, M17 metallo protease, bestatin

## Abstract

*Mycobacterium tuberculosis* is one of the most prevalent human pathogens causing millions of deaths in the last years. Moreover, tuberculosis (TB) treatment has become increasingly challenging owing to the emergence of multidrug resistant *M. tuberculosis* strains. Thus, there is an immediate need for the development of new anti-TB drugs. Proteases appear to be a promising approach and may lead to shortened and effective treatments for drug-resistant TB. Although the *M. tuberculosis* genome predicts more than 100 genes encoding proteases, only a few of them have been studied. Aminopeptidases constitute a set of proteases that selectively remove amino acids from the N-terminus of proteins and peptides and may act as virulence factors, essential for survival and maintenance of many microbial pathogens. Here, we characterized a leucine aminopeptidase of *M. tuberculosis* (MtLAP) as a cytosolic oligomeric metallo-aminopeptidase. Molecular and enzymatic properties lead us to classify MtLAP as a typical member of the peptidase family M17. Furthermore, the aminopeptidase inhibitor bestatin strongly inhibited MtLAP activity, *in vitro M. tuberculosis* growth and macrophage infection. In murine model of TB, bestatin treatment reduced bacterial burden and lesion in the lungs of infected mice. Thus, our data suggest that MtLAP participates in important metabolic pathways of *M. tuberculosis* necessary for its survival and virulence and consequently may be a promising target for new anti-TB drugs.

## Introduction

*Mycobacterium tuberculosis* is one of the most prevalent human pathogens. In the last year, an estimated 9.6 million people developed tuberculosis (TB) leading to 1.4 million deaths (WHO, 2016). In the last years, TB treatment has become increasingly challenging owing to the emergence of multidrug-resistant *M. tuberculosis* strains (Günther, 2014). Thus, there is an immediate need for the development of new drugs that target novel biological pathways to avoid cross-resistance. For instance, *M. tuberculosis* proteases appear to be a promising approach and may lead to shortened and effective treatments for drug-resistant TB (Roberts et al., 2013). Although the *M. tuberculosis* genome predicts more than 100 genes encoding proteases, only a few of them have been studied.

Proteolysis plays a central role in the biology of pathogens since it has been involved in their invasion, migration, acquisition of nutrients and evasion from inflammatory and immune responses.

Many studies have shown the importance of these proteins in bacterial (Ingmer and Brøndsted, 2009; Kennan et al., 2010; Frees et al., 2013), fungal (Xu et al., 2006; Yike, 2011), protozoan (Grellier et al., 2001; Klemba and Goldberg, 2002; Bastos et al., 2010; Suarez et al., 2013) and viral infections (Flexner et al., 2005; Konvalinka et al., 2015). Aminopeptidases constitute a diverse set of proteolytic enzymes that selectively remove amino acids from the N-terminus of proteins and peptides. Due to this kind of activity, aminopeptidases mediate, for example, the release of free amino acids to be used as a nitrogen source (Gonzales and Robert-Baudouy, 1996). Alternatively, these enzymes accomplish key steps in many activation or inactivation pathways by liberating amino acids from the N-terminus of self-derived proteins (Savijoki et al., 2006; Hearn et al., 2009; Naamati et al., 2009). Therefore, aminopeptidases act as virulence factors, essential for survival and maintenance of many microbial pathogens (Zhang et al., 2007; Skinner-Adams et al., 2010; Bhosale et al., 2012).

Aminopeptidases could be grouped according to the chemical nature of the catalytic site and substrate specificity (Rawlings and Barrett, 1993). One of the most intensively studied set of aminopeptidases is the hexameric leucine aminopeptidases (LAPs), which belongs to the M17 family of metalloproteases (Matsui et al., 2006). Found in animals, plants and microorganisms, LAPs play important roles in diverse physiological processes such as activation or inactivation of peptides, including neuropeptides and hormone peptides (Flexner et al., 2005), and trimming of MHC class I associated peptides in the endoplasmic reticulum (Naamati et al., 2009). In microbes, M17 LAPs have a function in proteolysis and have also acquired the ability to bind DNA (Matsui et al., 2006). Studies of M17 family members have increased in the last years due to their emergence as potential candidates for vaccine development and drug target in a number of parasitic and bacterial diseases (Acosta et al., 2008; Marcilla et al., 2008; McGowan et al., 2010; Carroll et al., 2012; Lee et al., 2015).

Leucine aminopeptidase is conserved and predicted to be essential for *in vivo* survival and pathogenicity of mycobacteria such as *M. tuberculosis, M. leprae, M. bovis, and M. avium paratuberculosis* (Ribeiro-Guimarães and Pessolani, 2007). Furthermore, there is increasing evidence that proteases are potential drug targets against bacterial infections such as tuberculosis (Roberts et al., 2013).

Herein, we describe the characterization of a LAPs of *M. tuberculosis* (MtLAP) as a cytosolic hexameric metallo-aminopeptidase. Moreover, molecular and enzymatic properties, such as susceptibility to inhibitors, lead us to classify MtLAP as a typical member of the peptidase family M17. The aminopeptidase inhibitor bestatin strongly inhibited MtLAP activity; *in vitro M. tuberculosis* growth, macrophage infection and it also reduced bacterial burden and lungs lesions in murine model of tuberculosis. Thus, our data suggest that MtLAP participates in important metabolic pathways of *M. tuberculosis* necessary for its survival and virulence and consequently may be a promising drug target.

## Materials and Methods

### Bacterial Strains and Growth Conditions

*Mycobacterium tuberculosis* H37Rv was grown at 37°C in Middlebrook 7H9 broth supplemented with 10% oleic acid-albumin-dextrose (OAD), 0.5% glycerol, and 0.05% Tween 80. *Escherichia coli* strain XL10-gold (Stratagene) was used for cloning and plasmid propagation. The recombinant protein was expressed in *E. coli* BL21 DE3 strain (Invitrogen). *E. coli* strains were maintained in Luria-Bertani (LB). Plasmid selection was performed with addition of kanamycin (50 μg/mL) or ampicillin (100 μg/mL) to the medium. Solid medium was prepared by the addition of 1.5% agar to the LB medium or 7H11 supplemented with 10% OAD.

### Mice

Male specific-pathogen-free C57BL/6 or interferon-gamma knockout (IFN-γ KO) mice aged 4–8 weeks, obtained from the animal care facility of the Institute of Tropical Pathology and Public Health at Federal University of Goiás (UFG), were maintained in isolators in class 3 biosafety level (BL3) cabinets with water and food provided *ad libitum*. Temperature was monitored and maintained around 22°C, relative humidity 60%, and 12 h light/dark cycles. The use of mice was conducted in accordance with the guidelines of the Brazilian Society of Animal Science Laboratory (SBCAL/COBEA). This study was carried out in accordance with the recommendations of Committee on the Ethics of Animal Experiments of Universidade Federal de Goiás (Approved protocol: 027/14).

### Molecular Cloning and Recombinant Protein Expression

*Mycobacterium tuberculosis* LAP gene (Rv2213^1^) was subcloned into PCR2.1 TOPO vector (Invitrogen) using a PCR amplified product from the *M. tuberculosis* H37Rv genome. The recombinant PCR2.1 vector was digested to release the gene that was ligated into the pET28a expression vector (Novagen), submitted to DNA sequencing and inserted into the expression host *E. coli* BL21. Bacteria containing the recombinant expression vector were grown at 37°C. When the bacterial cells reached OD_600_ measurements of 0.6, the expression of the recombinant protein was induced by the addition of isopropyl-beta-D-thiogalactopyranoside (IPTG) to a final concentration of 0.1 mM, and the incubation continued at 20°C during 16 h. Bacterial cells were collected by centrifugation (10,000 × *g* for 5 min), and suspended in 4 mL of binding buffer (20 mM imidazole, 0.5 M NaCl, and 20 mM Tris-HCl pH 7.9). After sonication, cell lysate was centrifuged (20,000 × *g* for 15 min), and the supernatant was applied onto a resin column (Novagen) charged with NiSO_4_ and equilibrated with binding buffer. Proteins were eluted with a concentration gradient of imidazole (40–000 mM) and protein concentration of the eluted fractions was determined by Bradford protein assay. The purified MtLAP was dialyzed against Tris-HCl pH7.5 with 250 mM NaCl and stored in 50% glycerol at -20°C. The purity of the protein was analyzed by Coomassie-stained 12% SDS-PAGE.

### Western Blot

H37Rv lysates and culture filtrate protein (CFP) were prepared as previously described Correa et al. (2014). Bacterial cultures (200 mL) were grown in Sauton media to late log phase and then pelleted by centrifugation at 2,000 × *g* for 20 min. Supernatant was transferred to a fresh tube, centrifuged again, and then filtered through a 0.2 μm filter unit. Supernatant was concentrated by protein acetone precipitation. Bacterial pellet was used for H37Rv lysates by sonication. Samples were resolved by 12% SDS-PAGE and transferred to nitrocellulose membranes. Membranes were blocked in 5% powdered milk in PBS and probed with the anti-MtLAP or anti-Zmp1 polyclonal serum produced in mice, followed by incubation with the anti-mouse IgG-alkaline phosphatase secondary antibody (Sigma–Aldrich). Antigen-antibody complex was detected via BCIP/NBT liquid substrate (Sigma–Aldrich).

### Analytical Ultracentrifugation

Sedimentation velocity experiments were performed using a Beckman XL-I analytical ultracentrifuge and an AN-60 TI rotor (Beckman Coulter). The experiments were carried out at 20°C for MtLAP at 18.2 μM in 25 mM Tris pH 7.5, 500 mM NaCl. A volume of 400 μL was loaded into 12 mm path cells and centrifuged at 130,000 × *g* (42,000 rpm). Scans were recorded every 3 min, overnight, at 280 nm. We used the Sednterp software^2^ to estimate the partial specific volume of the polypeptide chain, v ^-^ = 0.741 mL/g, the solvent density, ρ = 1.01920 g/mL, and the solvent viscosity, η = 1.0556 mPa.s, at 20°C. Sedimentation profiles were analyzed by the size-distribution analysis of Sedfit^3^. In Sedfit, finite element solutions of the Lamm equation for a large number of discrete, independent species, for which a relationship between mass, sedimentation and diffusion coefficients, s and D, is assumed, are combined with a maximum entropy regularization to represent a continuous size-distribution. We used 200 collected sets of data using fitted frictional ratio for sedimentation coefficients comprised between 0 and 45 S and a 0.68 confidence level.

### Assay of Enzyme Activity

The activity of MtLAP was assayed by incubating 300 ng of the purified enzyme with 20 μM of L-Leu-7-amido-4-methylcoumarin (Leu-AMC), Met-AMC, Pro-AMC, Arg-AMC, or Asp-AMC (Sigma–Aldrich) in 100 μL reaction buffer (25 mM Tris-HCl pH 7.5 and 1.5 mM NiSO_4_). Substrate hydrolysis was determined by AMC release measured fluorometrically using SpectraMax M5 Microplate Reader with SoftMax Pro Data Acquisition and Analysis Software (Molecular Devices) as described previously (Cadavid-Restrepo et al., 2011). Enzyme cation preference was performed using different concentrations of CaCl_2_, CoCl_2_, CuSO_4_, FeCl_2_, MgCl_2_, MnCl_2_, NiSO_4_, or ZnSO_4_ in Tris-HCl pH 7.5 at 25°C containing Leu-AMC as substrate. The optimal pH for activity was determined as described above in 25 mM AMT buffer (25 mM acetic acid-25 mM MES-25 mM Tris-HCl) adjusted to pHs ranging from 5 to 10. To assess the effect of temperature on MtLAP activity, enzyme reactions took place at temperatures ranging from 10 to 100°C. The Michaelis–Menten constant (Km) of MtLAP were determined according to the hyperbolic regression method using Prism software version 5.03 (GraphPad). The oligomeric structure of the enzyme was evaluated by electrophoresis as described previously (Cadavid-Restrepo et al., 2011). Briefly, purified protein was subjected to 8% SDS-PAGE in the presence 0.01% SDS under non-reducing conditions with or without previous boiling of the sample.

### MtLAP Inhibition

Different concentrations of tosyl-lysylchloromethane (TLCK), bestatin, tosyl phenylalanyl chloromethyl ketone (TPCK), EDTA, L-*trans*-epoxysuccinylleucylamido-4-guanidino butane (E-64), phenylmethylsulfonyl fluoride (PMSF), 1,10-phenanthroline, leupeptin, or pepstatin A were incubated with 300 ng of purified MtLAP in 100 μL reaction buffer for 5 min at room temperature, before the substrate was added. Enzymatic reactions were monitored as described above. All inhibitors were from Sigma–Aldrich. Bestatin half-maximal inhibitory concentration (IC50) and 95% confidence interval (CI) were calculated by non-linear regression log (inhibitor) vs. normalized response with variable slope method.

### Effect of Bestatin on *M. tuberculosis* Growth

To study the effect of bestatin on *M. tuberculosis* growth, bacterial cultures were adjusted, and 100 CFU were deposited into each well of a 96-well microplate that contained a serial dilution of bestatin, ranging from 1600 to 25 μg/mL in Middlebrook 7H9 supplemented with 10% OAD, 0.5% glycerol and 0.05% Tween 80. The bestatin 50% growth inhibitory concentration (GI50) value was determined by non-linear regression log (inhibitor) vs. normalized response with variable slope method.

### Alveolar Macrophage Culture

Alveolar macrophages were obtained from C57BL/6 mice bronchoalveolar lavage (BAL). Mice were euthanized and the pulmonary cavities were opened. Using an 18-gage needle, the trachea was cannulated, and 1.0 mL of ice-cold 5 mM EDTA in PBS was slowly injected into the lungs and then withdrawn. This procedure was repeated five times and a total of 4 mL of BAL fluid was collected from each mice. The single-cell suspension was then washed and centrifuged at 1,000 × *g*. Red blood cells were lysed upon 5 min incubation in 2 mL of Gey’s solution (155 mM NH_4_Cl and 10 mM KHCO_3_) at room temperature. Macrophages were then washed with PBS and resuspended in RPMI medium 1640 (Gibco) supplemented with 1% glutamine, 1% non-essential amino acids, 1 mM sodium pyruvate, 1% penicillin-streptomycin and 10% FBS.

### *M. tuberculosis* Infected Macrophage Treatment Assay

The alveolar macrophages were plated in 96 well plates (100 μL/well) at 10^6^ cells/well and incubated for 24 h at 37°C. Macrophage monolayers were then washed and incubated with 100 μL infection media (RPMI medium 1640 supplemented with 1% glutamine, 1% non-essential amino acids, 1 mM sodium pyruvate, 10% FBS) containing 5 × 10^6^ CFU of *M. tuberculosis* H37Rv (MOI = 5) at 37°C under CO_2_ for 3 h. Then the cells were washed twice with warm PBS to remove extracellular bacteria and supplemented with RPMI free of antibiotics and containing a range of 0, 100, or 200 μg/mL of bestatin. Forty-eight hours after infection, media was aspirated from infected macrophage wells, and 100 μL of ice-cold sterile lysis solution (0.05% SDS, w/v in H_2_O) was added to each well. The lysates were transferred to a new 96 well plate for serial dilutions. Triplicate wells were plated on 7H11 agar supplemented with OAD. The plates were cultured in a CO_2_ incubator for 3–4 weeks and the number of CFUs counted.

### *M. tuberculosis* Infection and *In vivo* Bestatin Treatment

IFN-γ knockout mice were intranasally infected with 10^4^ *M. tuberculosis* H37Rv and treatment was performed as previously described (Lenaerts et al., 2003). In brief, 1 day post infection, three mice were euthanized by cervical dislocation to verify bacterial uptake per mouse. Each treated group consisted of four mice. Treatment was started 18 days after infection and lasted up to 28 days post infection. Another three infected mice were euthanized to determine the bacterial load at the beginning of the treatment (day 18 post infection). Lyophilized bestatin was dissolved in PBS and 100 μL administered by intranasal instillation in eight treatments for 5 days/week at 1 mg/kg of body weight while Isoniazid (INH) was administered at 25 mg/kg as positive control. A negative control group of infected but untreated mice received 100 μL of intranasal PBS. After the cessation of treatment (day 28 post infection), viable bacteria in the mice lungs were evaluated. Right and left cranial lung lobes were removed and homogenized in PBS containing 0.05% Tween 80, and tissue homogenates were serially diluted in PBS and plated in duplicate onto Middlebrook 7H11 agar. Colonies were counted after 3–4 weeks of incubation at 37°C in a 5% CO_2_ incubator.

### Histopathology

The caudal left lung lobe was excised from all animals, stored in 10% formalin, then embedded in paraffin and sections were prepared and stained with hematoxylin and eosin (H&E). Lung lesions were determined using the measurement tool in the AxioVision software (Carl Zeiss). Total lesion area was calculated by the sum of all areas of lesions in a lung section per mouse.

### Statistical Analysis

Statistical significance between groups was determined by the two-tailed unpaired Student’s *t*-test or One-way ANOVA with Dunnett or Tukey post-test using Prism software version 5.03 (GraphPad).

## Results

### LAP Is Conserved in Pathogenic Mycobacteria

**FIGURE 1:**
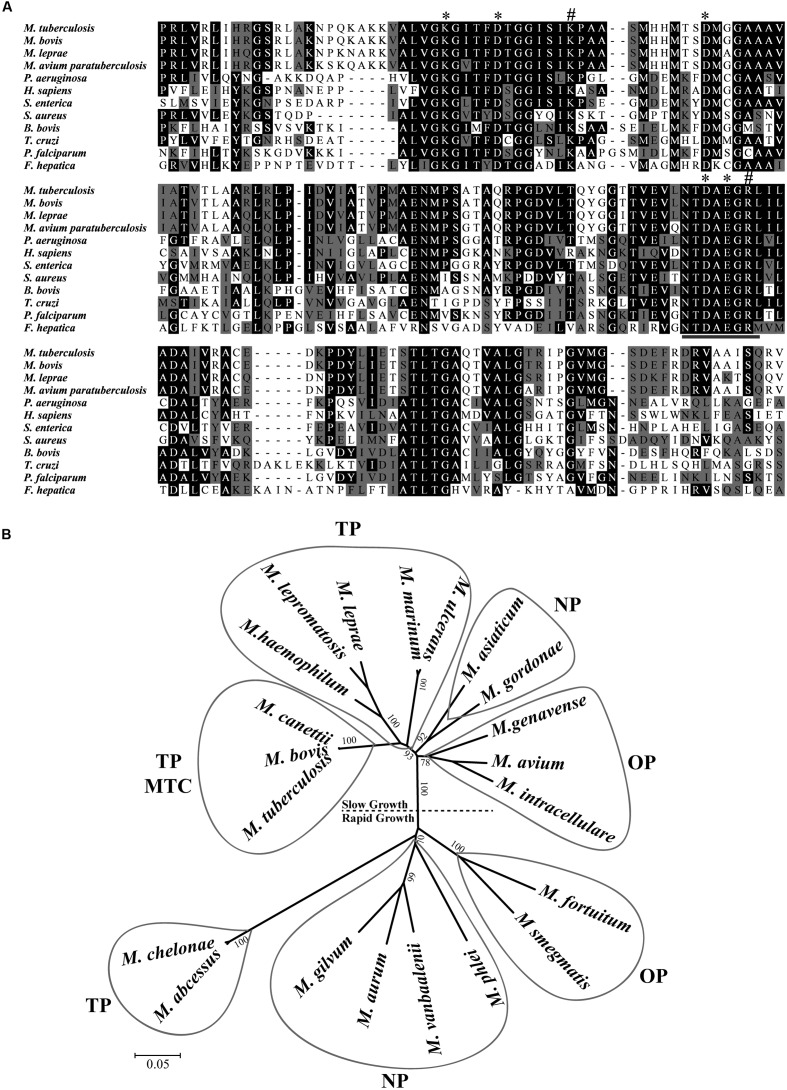
**Sequence analysis and phylogenetic relationship of M17 LAPs.** **(A)** Multiple Carboxi-terminal portion amino acid sequence alignments of different LAPs. Amino acid sequences from the conserved C-terminal region of LAPs were aligned by the ClustalX program. Amino acids marked in black show 50% identity and those in gray show 50% similarity. Putative metal binding sites (^∗^), catalytic site (#), and M17 signature (underlined) are indicated. Sequences were obtained from the protein database of the National Center for Biotechnology Information (NCBI). **(B)** Phylogenetic relationship of LAP in mycobacterial genus. 21 full length sequences, derived from the non-reduntant (NR) protein database of the NCBI (listed in Experimental Procedures), were aligned by the ClustalX program, and the phylogram generated with the Mega package after 1000 bootstraps inferred using the Neighbor-Joining method. The tree is drawn to scale with branch lengths in the same units as those of the evolutionary distances used to infer the phylogenetic tree. Drawn balloons represent the sequences clusters of true pathogenic (TP), *M. tuberculosis* Complex (MTC), opportunist pathogens (OP) and non-pathogenic mycobacteria (NP).

The sequencing of *M. tuberculosis* H37Rv genome revealed a small number of genes coding for putative peptidases that mediate aminopeptidolytic activities https://merops.sanger.ac.uk/index.shtml. We identified only one copy of a putative LAP gene (Rv2213^4^), which has thus far not been characterized. Rv2213 is an open reading frame (ORF) that encodes a protein of 515 amino acids. Multiple amino acid sequence alignments revealed that the C-terminal conserved region presents the typical signature sequence NTDAEGRL of the M17 family (Burley et al., 1990) as well as multiple conserved amino acids within predicted functional domains for metal binding and catalytic activity conserved across different taxa (**Figure 1A**). LAP of *M. tuberculosis* shares high identity with other pathogenic mycobacterial LAPs, over 80% with *M. leprae, M. bovis, and M. avium paratuberculosis.* Although it contains conserved functional domains, MtLAP shares less than 32% identity with species out of the *Mycobacterium* genus, diverging from LAPs from other bacteria, protozoa and higher eukaryotes, including only 28.1% identity with human LAP (**Figure 1A** and **Table 1**). The phylogenetic analysis of LAP from the *Mycobacterium* genus shows a relationship between LAP sequence similarities and bacterial physiology and pathogenesis. The phylogenetic tree shows divergent groups of proteins that cluster according to the bacterial rate of growth and pathogenicity with a strong bootstrap support (**Figure 1B**). Sequences are divided in two defined clades; one branch includes slow growing mycobacteria and the other cluster, the rapid growers. Moreover, for slow and rapid growing mycobacteria, LAP sequences are divided into three groups. One branch includes true pathogenic mycobacteria (TP), another branch groups opportunistic bacteria (OP) and the other branch contains non-pathogenic mycobacteria (NP). Similarly, true pathogenic slow growing mycobacteria belong to the *M. tuberculosis* complex (MTC) grouped together in a very close phylogenetic clade (**Figure 1B**). These findings suggest that MtLAP may be involved in physiological and pathogenicity processes in tuberculosis, thus predicted to be essential for *in vivo* survival and pathogenicity (Ribeiro-Guimarães and Pessolani, 2007).

**Table 1:** Leucine aminopeptidases (LAPs) amino acid sequence identity.

	Identity
	
Organism	*M. tuberculosis*
*M. bovis*	0.996
*M. leprae*	0.824
*M. avium*	0.805
*P. aeruginosa*	0.313
*H. sapiens*	0.281
*S. enterica*	0.265
*S. aureus*	0.258
*B. bovis*	0.253
*T. cruzi*	0.234
*P. falciparum*	0.211
*F. hepatica*	0.149


### MtLAP Is an Oligomeric Cytosolic Protein

**FIGURE 2:**
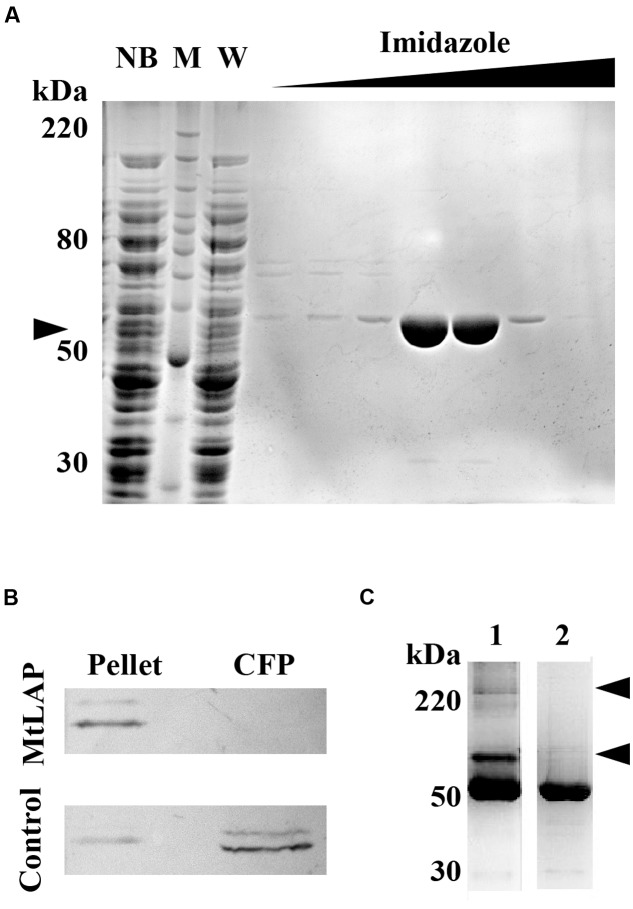
**Purification and subcellular localization of *M. tuberculosis* LAP (MtLAP).** **(A)** Purification of recombinant MtLAP: IPTG-induced MtLAP was purified by nickel affinity chromatography. Aliquots of resin unbound fraction (NB), molecular mass marker (M), wash (W) and elution with increasing imidazole concentration were submitted to 12% reducing SDS-PAGE and the gel stained with Coomassie brilliant blue. Arrowhead indicates LAP position at 55 kDa. **(B)** Localization of MtLAP by immunoblot analysis (up) and control of non-specific lysis (down). Lanes: H37Rv pellet total cell lysate (Pellet) and H37Rv culture filtrate proteins (CFP) were probed with MtLAP or Zmp1 (Control) polyclonal mouse antiserum. **(C)** Analysis of MtLAP oligomeric state: the recombinant enzyme was subjected to 8% SDS-PAGE in the presence of only 0.01% SDS, previously boiled (2) or not (1). Arrowheads indicate 100 kDa and over 220 kDa oligomeric states.

To establish the kinetic parameters of MtLAP, the *Mycobacterium tuberculosis* gene Rv2213 was cloned into the prokaryotic expression vector pET28a. The recombinant protein was expressed and purified by nickel affinity chromatography under non-denaturing conditions with increasing imidazole concentrations. SDS-PAGE analysis revealed a strong band, with high purity, eluted with imidazole at a concentration higher than 200 mM, corresponding to the expected protein molecular mass of approximately 55 kDa (**Figure 2A**). For detection and identification of the native protein, anti-MtLAP polyclonal serum was produced in mice. We verified by western blot the presence of the protein in the H37Rv cell fraction extract, but not in the culture supernatant (CFP, **Figure 2B**). To confirm that MtLAP was not released to the supernatant, mycobacterial metalloprotease Zmp1, previously described as a secreted protein (Master et al., 2008; Correa et al., 2014), was detected in the same culture supernatant preparations (**Figure 2B**). Since LAPs exist naturally as oligomeric-dependent active proteases (Matsui et al., 2006), we decide to test MtLAP oligomerization. Therefore, non-boiled nor reduced purified protein was subjected to 8% SDS-PAGE in the presence of 0.01% SDS. Bands about 100 kDa and over 220 kDa were detected indicating an oligomeric protein complex, although the 55 kDa monomer was predominant in gel (**Figure 2C**, lane 1). Sample boiling resulted in complete monomerization of MtLAP (**Figure 2C**, lane 2).

### MtLAP Assembles into a Homohexamer

**FIGURE 3:**
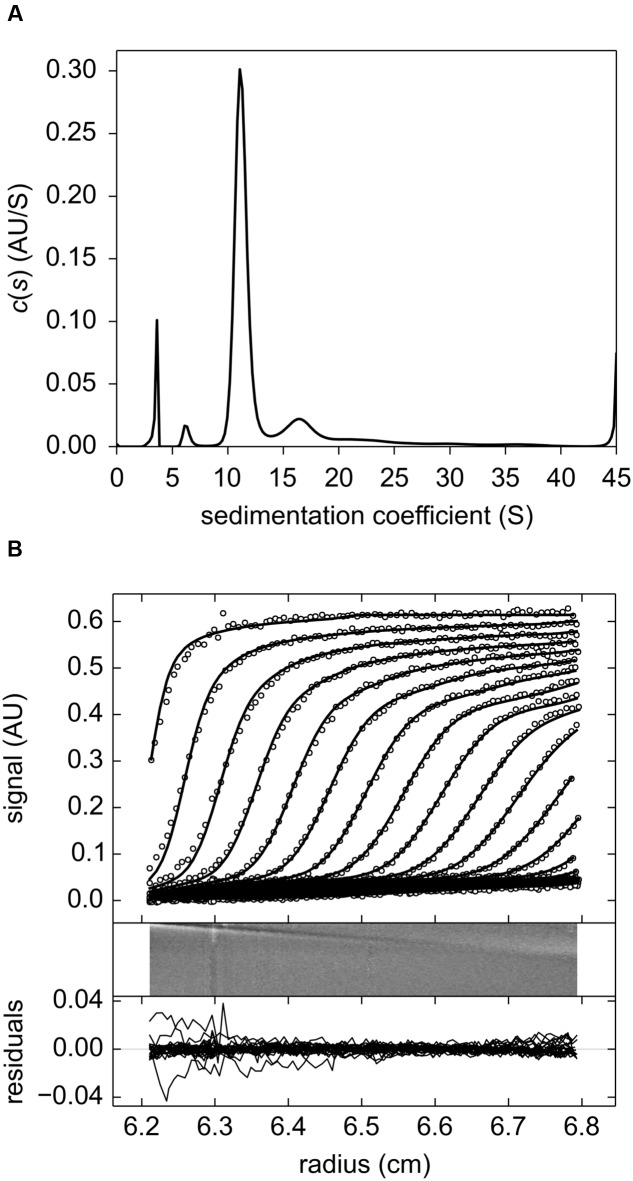
**Sedimentation velocity experiments of purified MtLAP.** **(A)** MtLAP experimental sedimentation profile at 18.2 μM in 25 mM Tris pH 7.5, 500 mM NaCl obtained at 280 nm at 130,000 × *g*, at 20°C, in 12 mm cell and their modeled profiles with the c(s) analysis (upper panel). The corresponding residual, superposition of the differences between the experimental and fitted curves (lower panel). **(B)** Result of the c(s) analysis for LAP at 18.2 μM.

The results obtained from sedimentation velocity experiments corroborate the calibrated exclusion size chromatography findings. The experimental and fitted sedimentation velocity profile of MtLAP (**Figure 3A**) obtained at 18.2 μM shows a major species, approximately 70% of the signal, that sediments at 11.2 S (S_20,w_ = 12.6 S). The *s*-value is directly related to the molar mass (*M*) and the Stokes radius (*R_S_*) of the particle. The combination of the *s*-values with R_S_ = 5.9 nm estimated from calibrated size exclusion chromatography gives an estimate for the MtLAP complex of *M* = 323 kDa, which is close to the expected molecular mass for a hexamer (334.4 kDa). Considering a hexamer, the R_S_ value corresponds to a frictional ratio of 1.30, a value close to 1.25 that relates to globular proteins. It suggests that the MtLAP hexamer has a globular shape. Larger species were also detected but a well-defined peak was difficult to identify. The larger species most probably represents aggregates due to sample aging and also to the fact that MtLAP was prone to precipitate even in high ionic strength buffers. The smaller peaks correspond to only less than 8% of the signal and give estimated masses of 64 and 140 kDa, close to the expected mass of a monomer and a dimmer, respectively (**Figure 3B**). The presence of these smaller forms may be related to the high salt concentration in the buffer. These data clearly indicate that active MtLAP is an oligomeric protein formed by six monomers.

### LAP of *M. tuberculosis* Is a Metal Dependent Protease

**FIGURE 4:**
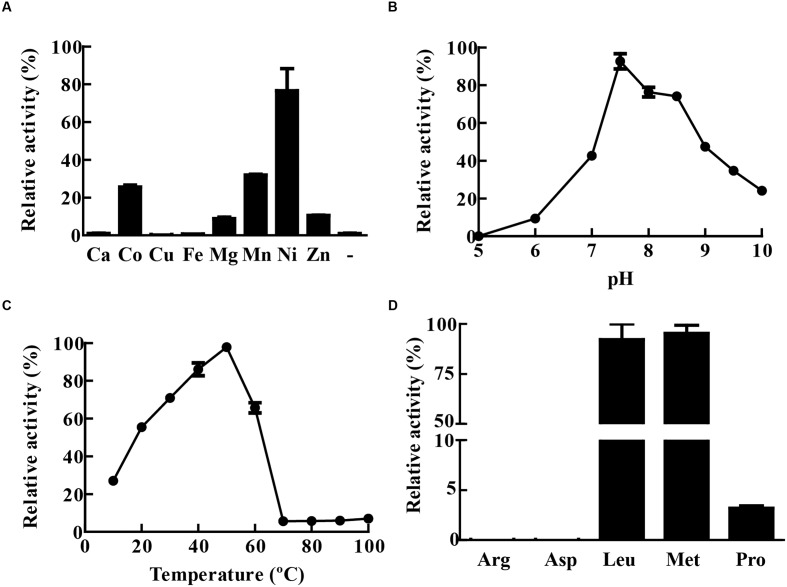
**Biochemical characterization of MtLAP.** **(A)** Effect of metal ions on MtLAP activity: Activity was assayed in the presence of different metal ions (1.5 mM concentration) and no metal addition (-). **(B)** Effects of pH on MtLAP activity: the experiments were performed over the pH range 5–10 in 25 mM AMT buffer with 1.5 mM NiSO_4_ and 20 μM substrate. **(C)** Effects of temperature on MtLAP activity: the experiments were carried out in 25 mM Tris–HCl buffer pH 7.5 over a temperature range of 10–100 °C with 1.5 mM NiSO_4_ and 20 μM substrate. **(D)** Activity of MtLAP against different amino acids: the experiments were carried out in 25 mM Tris–HCl buffer pH 7.5 with 1.5 mM NiSO_4_ and 20 μM of each indicate substrate. Data shown represent mean ± SEM, relative to maximal activity obtained, *n* = 3.

The activity of recombinant MtLAP was determined by measuring the fluorescence of AMC (7-amido-4-methylcoumarin) released by hydrolysis of the enzyme substrate Leu-AMC. However, in the initial assays, no MtLAP activity was observed when the enzyme was incubated with substrate in Tris-buffer in the absence of metal ions. As MtLAP belongs to the metallo aminopeptidase M17 family, that has a broad range of preferences for metal ions, we decided to test MtLAP metal dependence. The enzyme did show activity when the assay was conducted in the presence of metal ions including Co^2+^, Mg^2+^, Mn^2+^, Ni^2+^, and Zn^2+^. Conversely, Ca^2+^, Cu^2+^, and Fe^2+^ could not activate MtLAP. The maximum activity was observed in the presence of 1.5 mM Ni^2+^, followed by Mn^2+^, Co^2+^, Zn^2+^, and Mg^2+^ (**Figure 4A**). Regarding pH dependence, its optimal activity was observed at pH 7.5. Although at acidic pHs the enzyme rapidly loses activity and no substrate cleavage was observed at pH 5, the enzyme was resistant to alkaline environment displaying about 50 and 25% of its maximal activity at pH 9 and 10, respectively (**Figure 4B**). The activity of MtLAP was determined at different temperatures in the range of 10 – 100°C. At 37°C, the enzyme showed 70.78% of its maximal activity measured at 50°C (**Figure 4C**). To assess the enzyme substrate preference, MtLAP was incubated with aminopeptidase substrates (**Figure 4D**). Calculated *K*_m_ values are 69.4 ± 3.6 μM for Leu-AMC, 61.8 ± 4.4 μM and 344.8 ± 49.2 μM for Met-AMC and Pro-AMC, respectively (Supplementary Figure 1). The enzyme showed maximal activity on substrates with hydrophobic amino acids leucine and methionine. Low activity was observed with the non-polar cyclic amino acid proline based substrate. No cleavage was detected on substrates with charged side chains containing arginine and aspartic amino acid residues.

### MtLAP Inhibition Pattern

**FIGURE 5:**
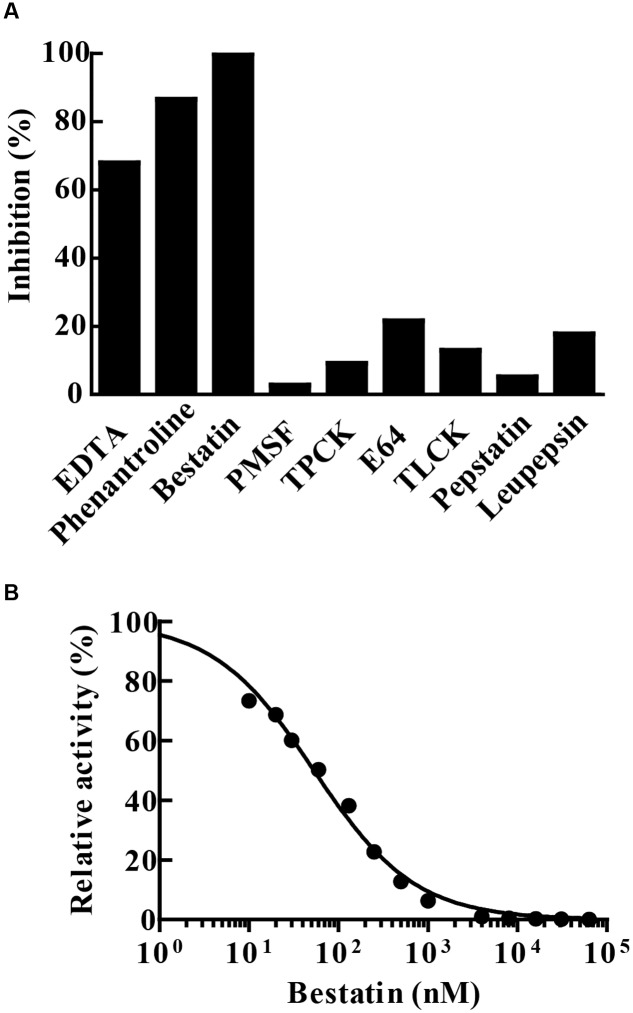
**MtLAP inhibition pattern.** **(A)** Effect of different inhibitors on the activity of MtLAP: the experiments were performed in 25 mM Tris–HCl buffer pH 7.5 with 1.5 mM NiSO_4_, 20 μM of Leu-AMC and different inhibitors (10 mM EDTA, 10 mM phenantroline, 30 μM bestatin, 5 mM PMSF, 100 μM TPCK, 5 μM E64, 500 μM TLCK, 25 μM pepstatin A and 100 μM leupepsin). Data represent % of the activity measured without inhibitors. **(B)** Bestatin inhibits the enzymatic activity of MtLAP in a dose-dependent manner: the experiments were carried out by pre-incubation of MtLAP with different concentrations of bestatin. Curve fit by non-linear regression log (inhibitor) vs. normalized response with variable slope method.

Enzyme hydrolytic activity was not sensitive to the classical serine (PMSF, TLCK or TPCK) and cysteine (E-64) protease inhibitors. The enzyme was not inhibited by leupeptin, an inhibitor of cysteine/serine/threonine proteases, nor by pepstatin A that inhibits aspartyl proteases. The chelating agents 1,10-phenanthroline and EDTA inactivated 86.5 and 68% of the enzyme activity, respectively. Furthermore, the enzymatic activity of MtLAP on Leu-AMC was completely inhibited by 30 μM bestatin (**Figure 5A**). Since bestatin is a potent inhibitor of LAPs, we assayed its inhibition on MtLAP activity in a range of 0.01–100 μM using Leu-AMC as a substrate (**Figure 5B**). As a result, the calculated IC_50_ was 53.78 nM as shown in **Figure 5B** with 95% CI of 46.44–62.28 nM.

### Bestatin Inhibits *M. tuberculosis* Growth in *In vitro* and *Ex vivo*

**FIGURE 6:**
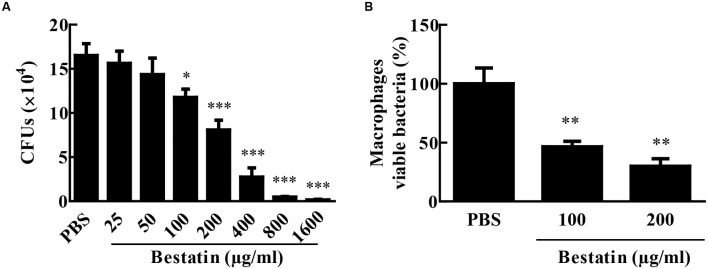
**Bestatin inhibits growth of *M. tuberculosis in vitro* and during macrophage infection.** **(A)** Bestatin inhibits the growth of *M. tuberculosis in vitro* in a dose-dependent manner: *M. tuberculosis* was incubated with or without (PBS) different concentrations of bestatin at 37°C, and growth was monitored 7 days after addition of the inhibitor by plating serial dilution of culture and counting CFUs. **(B)** *M. tuberculosis* survival in macrophages: After macrophage infection, bestatin or PBS was added to the culture and viable *M. tuberculosis* was determined by CFU counting relative to the PBS group 48 h after treatment. Data shown represent mean ± SEM, n = 3. ^∗^*p* < 0.05; ^∗∗^*p* < 0.01; ^∗∗∗^*p* < 0.001; difference from PBS group by One-way ANOVA with Dunnett post test.

Since bestatin is efficient at inhibiting MtLAP activity, we asked if it could constrain *M. tuberculosis* growth. To address this question, we cultivated the bacteria in liquid media with different concentrations of bestatin and measured CFUs. At day seven of culture, a dose-dependent manner inhibition of *M. tuberculosis* growth was observed (**Figure 6A**). Bestatin shows a 50% growth inhibitory concentration value (GI50) of 174.2 μg/mL (95% CI 143.1–212.0 μg/mL). We next examined the effect of bestatin on *M. tuberculosis* survival inside macrophages. The bestatin treatment done after *M. tuberculosis* alveolar macrophages infection showed a significant dose-dependent reduction in colony counts. Compared to PBS infection control, 29.8% of the mycobacteria were recovered using 200 μg/mL of inhibitor and 46.4% of the bacteria were viable using bestatin at 100 μg/mL (**Figure 6B**). Those results suggest that the activity of LAP may be essential for *M. tuberculosis* survival and virulence.

### Bestatin Reduces *In vivo* Bacterial Burden and Lung Lesions

**FIGURE 7:**
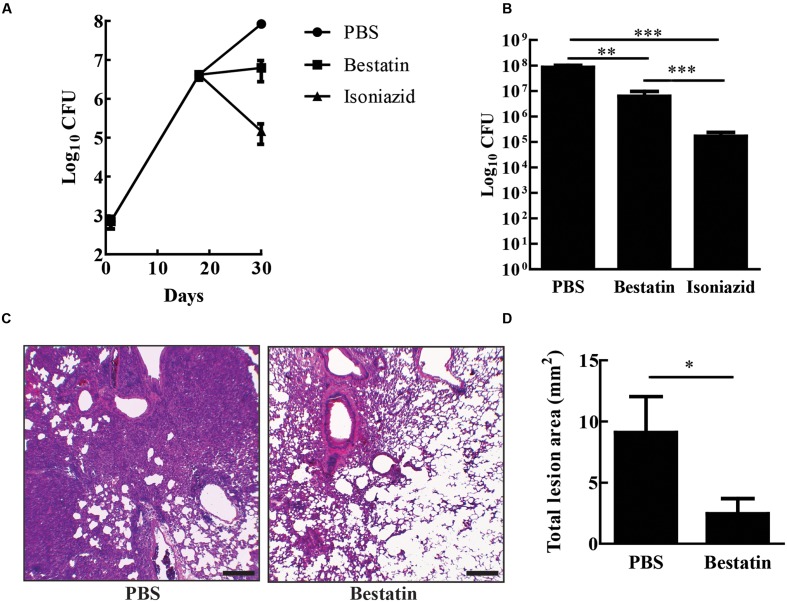
**Bestatin reduces *in vivo M. tuberculosis* burden and lung lesion.** **(A)** IFN-γKO mice were intranasal infected with 10^4^ CFU of *M. tuberculosis* H37Rv. Eighteen days after infection, the mice were treated with bestatin at 1 mg/kg (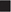) isoniazid (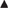) and control group of infected mice were treated with PBS (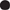). The bacillary loads in the lungs were determined after euthanizing the mice at days 1, 18, and 28 post infections. **(B)** Comparison of lung CFU after treatment. **(C)** Representative lung sections (left bottom lobe) with HE stain of *M. tuberculosis* infected mice treated with PBS (left) or bestatin (right). Scale bars represent 200 μm. **(D)** Results of the quantification of the inflammatory lesion area sizes. Data shown represent mean ± SEM, *n* = 4. ^∗^*p* < 0.05; ^∗∗^*p* < 0.01; ^∗∗∗^*p* < 0.001; difference between groups by One-way ANOVA with Tukey’s Multiple Comparison Test or *t*-test.

Bestatin has been used before as a low molecular immunomodulator. It exhibits antitumoral and antimicrobial activities through the activation of host defense mechanisms and shows low toxicity even after a long-term administration (Ota, 1991). In several mouse-model studies, effective and safe bestatin doses ranged from 0.1 to10 mg/kg of body weight or in doses of 10–100 μg per animal (Bruley-Rosset et al., 1979; Carter et al., 2012; Reinhoudt et al., 2012; Lis et al., 2013). To test whether bestatin has any effect on tuberculosis progression, IFN-γ knockout mice were infected with 10^4^ CFU of *M. tuberculosis* H37Rv and treated with the inhibitor at 1 mg/kg/day intranasally. After completion of therapy, bestatin reduced bacterial burden (∼1.4 Log) in the lungs when compared to PBS administration and isoniazid corroborates the efficacy of treatment method (**Figures 7A,B**). Thus, bestatin inhibits up to 95.37% of *M. tuberculosis* growth in the mice lungs. In addition, treatment reduced lung lesions caused by infection (**Figure 7C**) from 9.09 mm^2^ of lung lesion area in PBS group to 2.46 mm^2^ in bestatin treated mice (**Figure 7D**). Taken together, our results indicate that bestatin is a promising drug and a model compound for anti-TB drugs.

## Discussion

Leucine aminopeptidases are known to play important roles in bacterial physiology and have not yet been characterized in many pathogenic bacteria, including *M. tuberculosis*. In this study we report the identification and biochemical characterization of the *M. tuberculosis* M17 family LAP as a cytosolic metal-dependent protease that shows high sensitivity to bestatin. In addition, *M. tuberculosis in vitro* growth and survival in macrophages had a significant bestatin dose-dependent reduction. Furthermore, bestatin treatment in murine tuberculosis model was useful in reducing both mycobacterial load in the lungs and tissue lesions caused the infection.

Aminopeptidases are widely distributed in animals, plants and microorganisms, and found in the extracellular millieu, cytoplasm, in many subcellular organelles and as components of membranes (Matsui et al., 2006). M17 protease members produced by a number of pathogenic organisms are currently under intense investigation. In some of these cases, the enzyme is secreted and act extracellularly. However, bacterial LAPs are most commonly found in the cytosol and generally act as homo-hexameric enzymes, while extracellular LAPs are functional as monomers (Gonzales and Robert-Baudouy, 1996). In agreement with literature, we found that the *M. tuberculosis* LAP is cytosolic (**Figure 2B**) and probably acts as an oligomer enzyme (**Figure 2C**) formed by six monomers (**Figure 3**). The enzyme could otherwise be secreted *in vivo* under specific physiological conditions.

Leucine aminopeptidases belong to the M17 family of metalloproteases, which mainly prefer Zn^2+^, while other aminopeptidases are dependent on Mn^2+^, Fe^2+^, or Mg^2+^. Like that of *Staphylococcus aureus* (Singh et al., 2012), *M. tuberculosis* LAP exhibits maximum activity in the presence of Ni^2+^, but it is active in the presence of a broad range of other metals (**Figure 4A**). The wide-ranging metal cofactor profile of LAP may allow its activity under different *in vivo* conditions, where some metals are limited and tightly regulated by both host and bacteria, resulting in an altered enzymatic activity under certain conditions (Kehl-Fie et al., 2011; Hood and Skaar, 2012).

Despite conservation of amino acid sequences, M17 members show variable pH and temperature optima. Although MtLAP is active over a broad range of temperatures with optimal 55°C, at physiological temperature the enzyme remains with high activity (**Figure 4C**). Moreover, its activity shows a marked dependence on neutral/alkaline pH, since the enzyme is completely inactive at pH 5 (**Figure 4B**). This correlates well with observations that recombinant members of M17 proteases assemble better into active oligomers at higher temperature and alkaline pHs (Matsui et al., 2006; Cadavid-Restrepo et al., 2011). Thus, the ability of *M. tuberculosis* to preserve intrabacterial pH inside phagosomal compartments (Vandal et al., 2008) provides an optimum environment for MtLAP function during infection. MtLAP does cleave substrates with charged side chains and shows preference for substrates with hydrophobic amino acids (**Figure 4D**). This substrate specificity profile supports future search for specific inhibitors of *M. tuberculosis* LAP, which could be used as new TB drugs.

Bestatin is a natural dipeptide analog product isolated from actinomycetes. It potently inhibits aminopeptidases, including LAP M17 family (Suda et al., 1976; Burley et al., 1991; Matsui et al., 2006), and MtLAP shows high sensitivity to this inhibitor (**Figures 5A,B**). Bestatin inhibited MtLAP enzymatic activity as well as *M. tuberculosis in vitro* growth, suggesting that MtLAP activity is associated to metabolism and physiology of the bacteria (**Figure 6A**). Moreover, bestatin has been shown to inhibit *in vitro* growth of *S. aureus* (Singh et al., 2012) and *Babesia bovis* (Aboge et al., 2015), as well as *P. falciparum* in mouse models of malaria (Nankya-Kitaka et al., 1998; Gavigan et al., 2001; Naughton et al., 2010). The reduction mediated by bestatin of bacterial burden and lung tissue lesions lead by infection, (**Figure 7**), suggests MtLAP may act not only in bacterial physiology but also in *M. tuberculosis* virulence. These results support further experiments to establish the role of MtLAP in the pathogenesis of TB. Bestatin is associated with immunomodulatory effects activating monocytes/macrophages and promoting the secretion of the pro-inflammatory cytokines IL-1, IL-6, IFN-γ, and TNF-α, as well as the growth factors GM-CSF and G-SCF by immune system cells (Müller et al., 1982; Schorlemmer et al., 1983; Shibuya et al., 1987; Okamura et al., 1990). It has been demonstrated that bestatin can be administered to animals at very low toxicity and with multiple positive effects on the immune system. Those studies have also revealed beneficial effects on the survival of animals with some experimental tumors. Subsequent research in human patients have confirmed the immunomodulatory effects of bestatin and provided encouraging results on the potential of this drug in cancer treatments (Talmadge et al., 1986; Inoi et al., 1995). Bestatin is used as an immunomodulator and antitumor drug, under the trademark Ubenimex and various active stereoisomers and substituted analogs are commercially available as useful tools for *in vitro* and animal drug experimentation (Ota, 1991; Scornik and Botbol, 2001). Moreover, bestatin may have effects direct on bacteria and/or in modulation of host immune system leading to bacterial control. Thus, more studies are needed to establish whether bestatin mechanism of action actually correlates with mycobacterial proteins beyond LAP or even with host enzymes.

In toxicological studies, bestatin showed very low toxicity and no death occurred following the oral administration of the maximum dose capable of dosing such as 4 g/kg for mice, 2 g/kg for rats and 1.2 g/kg for dog. General toxic signs seen in mice and rats following subcutaneous and intraperitonial injections were depression, suppressed movement, piloerection, inhibition of spontaneous movement, anorexia and emaciation (Sakakibara et al., 1983). In human clinical studies of bestatin, hematology, hepatic and renal function tests revealed no apparent adverse reactions and minimal side effects were noted chiefly comprising of reversible gastro-intestinal disturbances (Saito et al., 1983; Ikeda et al., 1985; Tsukagoshi, 1987; Ichinose et al., 2003). Although bestatin properties seem to be promising, pharmacokinetics could be an obstacle to its use as a TB drug. In mice, ^3^H-bestatin diffuses rapidly from the intravascular space into the extracellular fluid after its intravenous injection and from there most of the drug is eliminated in the urine (Scornik and Botbol, 1997). Furthermore, bestatin, orally administered, is efficiently absorbed by the intestinal brush border, whereas high concentrations of the drug in the serum are maintained only for a short time in mice because of its rapid elimination (Abe et al., 1989). Otherwise, urinary elimination is slower in humans (Ueda et al., 1994). Moreover, intrapulmonary delivery of isoniazid, capreomycin, or amikacin to mice has resulted in a better reduction of pulmonary mycobacterial loads than that seen by standard drug delivery methods when lower concentrations of drugs and fewer doses have been used (Gonzalez-Juarrero et al., 2012; Pham et al., 2015). Thus, in our study, intranasal administration of bestatin may have extended drug effects in the lungs, the main TB target organ, bypassing the problem of its rapid urinary elimination and thus showing efficacy to reduce *M. tuberculosis* loads in mice lungs. The bacterial counts in infected mice indicates that bestatin probably has *in vivo* bacteriostatic activity since it has maintained the pulmonary bacterial load constant during the time course of the treatment whereas isoniazid decreases the *M. tuberculosis* burden in the mice lungs (**Figure 7A**). Taking the above facts with our findings, bestatin or its analogs that show/have better pharmacokinetics could be promising drugs on TB treatment.

In summary, our results showed that *M. tuberculosis* produces an oligomeric cytosol LAPs that is a member of the M17 metalloprotease family. The activity of *M. tuberculosis* LAP is very sensitive to bestatin, a powerful aminopeptidase inhibitor. In addition, bestatin effects on mycobacterial growth and TB mice model infection highlight aminopeptidases as potential drug targets in tuberculosis.

## Author Contributions

Conceived and design the experiments: JdS and AC; Performed the experiments: AC and DN; Analyzed the data: AC, JdS, DN, and IB; Contributed reagents/materials/analysis tools: AJ-K, IB, AK, JdS; Wrote the manuscript draft: AC; Critically revised the manuscript: All authors.

## Conflict of Interest Statement

The authors declare that the research was conducted in the absence of any commercial or financial relationships that could be construed as a potential conflict of interest.

## Footnotes

1 http://genolist.pasteur.fr/TubercuList

2 http://www.jphilo.mailway.com/

3 http://www.analyticalultracentrifugation.com

4 http://tuberculist.epfl.ch
