# Supplementary material for: The Activity of a Hexameric M17 Metallo-Aminopeptidase Is Associated With Survival of Mycobacterium tuberculosis
Source: Front Microbiol. 2017 Mar 27;8:504. doi: 10.3389/fmicb.2017.00504 (PMC5366330; doi:10.3389/fmicb.2017.00504)
Supplement: Supplementary file 1 [file Image_1.pdf]

Supplementary Material

The activity of a hexameric M17 metallo-aminopeptidase is associated with survival of *Mycobacterium tuberculosis*

Andre França Correa[1,2], Izabela Marques Dourado Bastos[1], David Neves[1], Andre Kipnis[2], Ana Paula Junqueira-Kipnis[2] and Jaime Martins de Santana[1,*]

Laboratório de Interação Patógeno-Hospedeiro, Instituto de Biologia, Universidade de Brasília, Brasília, Brazil

Instituto de Patologia Tropical e Saúde Pública, Universidade Federal de Goiás, Goiânia, Brazil

***Correspondence:**

Jaime Martins de Santana jsantana@unb.br

Supplementary Figures

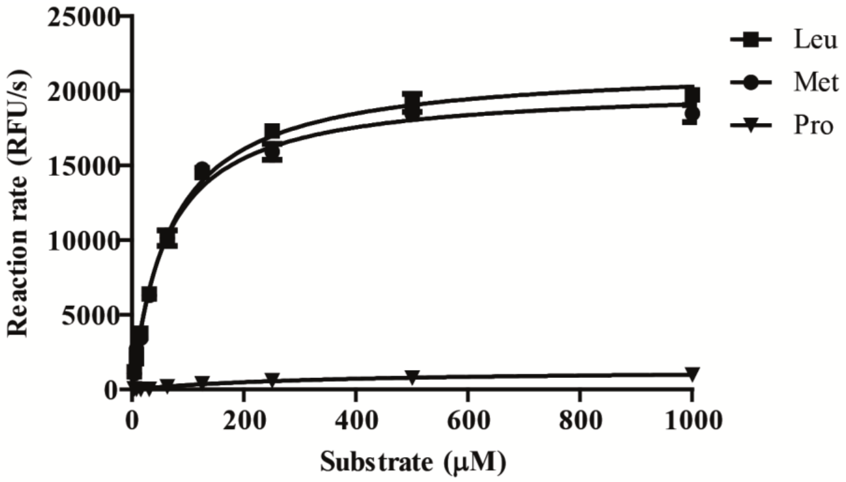

Supplementary Figure 1. Nonlinear regression fit curve for activity of MtLAP on substrates Leucine-AMC (■), Methionine-AMC (●) and Proline-AMC (▼). The Michaelis-Menten constant (Km) of MtLAP were determined according to the hyperbolic regression method using Prism software version 5.03 (GraphPad).
